# Retrograde intrarenal surgery for treatment of stones in congenital anomalous kidneys: a case-matched comparative study with extracorporeal shockwave lithotripsy

**DOI:** 10.1007/s00240-025-01860-8

**Published:** 2025-10-06

**Authors:** Abul-fotouh Ahmed, Mahmoud ElMesery, Mohamed Algammal, Abdullah Daoud, Ahmed Solyman, Ahmed Mosaad

**Affiliations:** Department of Urology, Faculty of Medicine, Al-Azhar University, Cairo, 11633 Egypt

**Keywords:** Congenital renal anomalies, Kidney stones, Retrograde intrarenal surgery, Shockwave lithotripsy, Stone-free rate

## Abstract

**Objectives:**

To compare the efficacy and safety of extracorporeal shockwave lithotripsy (SWL) and retrograde intrarenal surgery (RIRS) in the management of renal stones in patients with congenital renal anomalies.

**Methods:**

In this matched-group comparative study, we prospectively enrolled patients with congenital renal anomalies and renal stones ≤ 20 mm who underwent RIRS. These patients were matched 1:1 with those who underwent SWL at our institution. The primary outcome was the stone-free rate (SFR). Secondary outcomes included operative time, fluoroscopy time, complication rates, hospital stay, and need for retreatment.

**Results:**

A total of 166 patients were included (83 in each group). Baseline demographic, clinical, and stone characteristics were comparable between the two groups, except that patients in the RIRS group had significantly higher BMI and stone radiodensity. The distribution of anomalies was as follows: malrotation (79.5%), horseshoe kidney (6.0%), pelvic kidney (10.8%), duplex system (2.4%), and crossed renal ectopia (1.2%). RIRS achieved a significantly higher SFR than SWL (81.9% vs. 56.6%, *p* < 0.001). Complication rates were comparable between the groups (RIRS: 24.1%, SWL: 20.5%), with the majority being minor (Clavien–Dindo grade I–II). RIRS was associated with a longer operative time, shorter fluoroscopy exposure, and significantly lower retreatment rates (19.3% vs. 85.5%, *p* < 0.001). The median hospital stay was 2 days for RIRS, while SWL was performed on an outpatient basis.

**Conclusion:**

In patients with congenital renal anomalies, RIRS provides superior stone clearance and significantly reduces the need for retreatment compared to SWL, while maintaining a comparable safety profile.

**Trial registration:**

The trial was registered at ClinicalTrials.gov (NCT05240170) (04/02/2022).

## Introduction

The management of renal stones in patients with congenital renal anomalies presents significant challenges due to altered renal anatomy, including abnormal calyceal orientation, ectopic positioning, and anomalous vascular patterns [1–3]. While current guidelines offer evidence-based recommendations for stone management in anatomically normal kidneys, there is no consensus regarding optimal treatment in anomalous kidneys [4].

Available treatment modalities include open surgery, percutaneous nephrolithotomy (PNL), extracorporeal shockwave lithotripsy (SWL), and retrograde intrarenal surgery (RIRS). Open surgery, although associated with high stone-free rates (SFR), is highly invasive and rarely employed in contemporary practice due to significant morbidity. PNL is effective but carries increased risks in anomalous kidneys, particularly due to aberrant anatomy and proximity to adjacent structures [5, 6]. Consequently, minimally invasive approaches like SWL and RIRS are often preferred.

SWL is non-invasive and generally well tolerated but offers modest SFRs, often requiring multiple sessions or auxiliary procedures. Its efficacy is further limited in anomalous kidneys by difficulties in stone localization and impaired drainage of fragments [7–9]. RIRS, facilitated by advances in endoscopic technology, has emerged as a promising alternative, providing higher SFRs with favorable safety profiles [5, 6, 10]. Nevertheless, existing comparative studies are limited by retrospective design, small cohorts, and lack of matched controls.

In this prospective matched-group study, we evaluated the safety and efficacy of RIRS versus SWL for stone treatment in kidneys with congenital anomalies. The findings aim to provide clearer guidance on optimal management strategies for this unique patient population.

## Patients and methods

### Study design and patients’ population

This open-label, interventional, matched-group comparative study was conducted at a tertiary care academic center in Egypt between April 2022 and November 2024. Ethical approval was obtained from the institutional review board, and the study was registered with ClinicalTrials.gov (NCT05240170). Informed consent was obtained from all prospectively enrolled participants.

Adult patients with congenital renal anomalies and renal stones measuring ≤ 20 mm were prospectively enrolled in the RIRS group. These patients were matched 1:1 with a historical control group who had undergone SWL at the same institution. Matching was based on the type of renal anomaly and stone location (lower pole vs. non-lower pole).

Exclusion criteria included pregnancy, severe orthopedic deformities, uncorrectable coagulation disorders, active urinary tract infection (UTI), stones located within calyceal diverticula, urinary tract obstruction distal to the stone, or concomitant pathology requiring simultaneous surgical intervention.

Using G*Power 3.1 software, the sample size was calculated based on previously reported SFRs for RIRS and SWL in anomalous kidneys [5, 6, 8, 10–13]. Assuming a 15% effect size, 80% power, and a 5% alpha error, the required sample size was 164 patients (82 per group). Accounting for potential dropouts, the final target sample size was set at 174 (87 per group).

### Study procedures

In the RIRS group, all patients underwent preoperative comprehensive clinical and laboratory assessment, including urinalysis, urine culture, complete blood count (CBC), serum creatinine, liver function tests, and coagulation profile. Imaging studies included plain abdominal radiography (KUB), ultrasonography, and non-contrast computed tomography (NCCT). Contrast imaging was selectively employed for detailed anatomical evaluation when necessary.

The procedure was performed under general or spinal anesthesia by experienced endourologists. A flexible ureteroscope (OUT Medical) and holmium: YAG laser were used for lithotripsy. A ureteral access sheath (UAS) was employed when feasible. In cases of tight ureters, a double-J stent was inserted, and the procedure was postponed for two weeks. At the end of the procedure, a stent was placed if clinically indicated.

Postoperative evaluations on the first day included clinical assessment, KUB X-ray, abdominal ultrasonography, and CBC. Follow-up evaluations at two and four weeks comprised clinical assessment, urinalysis, CBC, serum creatinine measurement, and abdominal ultrasonography. NCCT was performed at the two-week follow-up visit, and repeated if indicated. The double-J stents were removed after 2 weeks if no significant residual fragments were present. Repeat RIRS was scheduled in cases with clinically significant residuals.

In the SWL group, all procedures were performed using the Dornier SII lithotripter. In accordance with our institutional policy, preoperative diagnosis was established with NCCT. Follow-up imaging was performed after each SWL session, and the decision to proceed with additional sessions was determined by the presence of residual stone burden assessed by ultrasonography, KUB, or NCCT when necessary. If residual fragments > 4 mm were detected, repeat SWL was scheduled, with a maximum of three sessions. The final assessment of stone-free status was based on NCCT. The collected data included demographic details, stone characteristics, analgesia method, imaging modality used for stone localization, the number of shockwaves delivered, and the number of treatment sessions. Follow-up assessments recorded stone-free status, complications, need for auxiliary procedures, and unplanned healthcare visits.

### Study outcomes

The primary endpoint was the SFR, defined as the absence of residual stones or the presence of clinically insignificant fragments (< 4 mm) on NCCT within 4 weeks post-treatment. Secondary endpoints included operative time, fluoroscopy time, length of hospital stay, rates of retreatment and auxiliary procedures, unplanned admissions, the need for blood transfusion, postoperative complications, and direct medical costs.

Operative time was defined as the duration from the induction of anesthesia to the placement of the urethral catheter for RIRS, and from the initiation of analgesia or sedation to the completion of the session for SWL.

Costs were calculated based on internal hospital pricing records and included all essential elements: preoperative evaluations, anesthesia, medications, procedural expenses (including equipment and disposables), postoperative care, and hospital stay.

### Statistical analysis

Data analysis was performed using SPSS version 26.0 (IBM Corp., Armonk, NY, USA). Continuous variables were expressed as mean ± standard deviation (SD) or median (interquartile range [IQR]) depending on data distribution. Categorical variables were presented as frequencies and percentages. Intergroup comparisons were made using the independent-sample *t*-test or Mann–Whitney *U* test for continuous variables and the chi-square or Fisher’s exact test for categorical variables. A *p*-value < 0.05 was considered statistically significant.

## Results

**Fig. 1:**
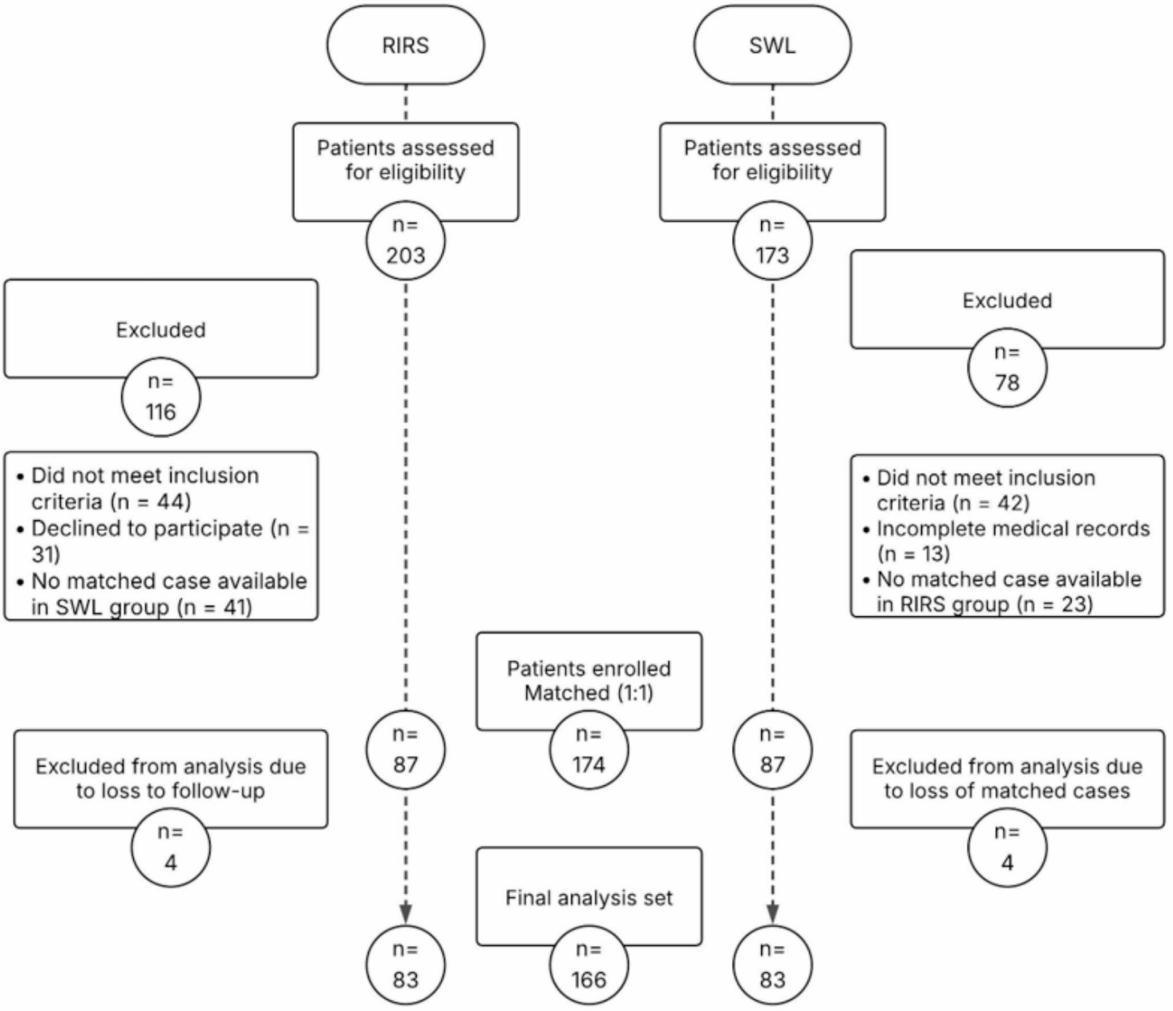
Flow diagram of patient selection and inclusion in the RIRS and SWL groups. RIRS, Retrograde Intrarenal Surgery; SWL, Shockwave Lithotripsy

A total of 174 patients were enrolled, with 87 patients allocated to each group. Four patients in the RIRS group were lost to follow-up, and their matched SWL counterparts were excluded, yielding a final cohort of 166 patients (83 per group). The patient selection process is outlined in Fig. 1.

The median age was 44.5 years (IQR 19.5), and the median BMI was 26.6 kg/m² (IQR 4.2). Patients in the SWL group were significantly older (*p* = 0.001), whereas the RIRS group had a significantly higher BMI (*p* < 0.001). The most common renal anomaly was simple malrotation (55.4%). Most stones were solitary (97.0%) and located in non-lower pole calyces or renal pelvis (77.1%). The median stone size was 12.0 mm (IQR 6.0). Radiodensity was significantly higher in the RIRS group (*p* = 0.001). Baseline characteristics are summarized in Table 1.

**Table 1:** Baseline data

Variables	RIRS group (*n* = 83)	SWL group (*n* = 83)	*p*-value
Age (yrs), median (IQR)	38.0 (32–49.8)	47.0 (40.3–57.5)	0.001
Gender, n (%)			0.061
Males	70 (84.3)	59 (71.1)	
Females	13 (15.7)	24 (28.9)	
BMI, median (IQR)	27.8 (26.3–31.4)	26.1 (25.2–27.8)	< 0.001
Previous stone-related intervention, n (%)	26 (31.3)	22 (26.5)	0.493
Hemoglobin, g/dL, mean (SD)	12.9 ± 1.7	12.6 ± 1.5	0.359
S. creatinine, mg/dL, mean (SD)	1.1 ± 0.4	1.0 ± 0.3	0.129
Renal anomalies, n (%)			–
Malrotated	46 (55.4)	46 (55.4)	
Horseshoe	13 (15.7)	13 (15.7)	
Ectopic	19 (22.9)	19 (22.9)	
Duplicated	5 (6.0)	5 (6.0)	
Operated side, n (%)			0.275
Right	34 (41.0)	41 (49.4)	
Left	49 (59.0)	42 (50.6)	
Pelvicalyceal dilation	38 (45.8)	40 (48.2)	0.756
*Degree*			
Mild	25 (65.8)	32 (80.0)	0.157
Moderate	13 (34.2)	8 (20.0)	
Stone size (mm), median (IQR)	12.2 (10.0–17.0)	12.0 (10.0–14.0)	0.227
Stone location, n (%)			–
Lower polar	19 (22.9)	19 (22.9)	
Non-lower polar	64 (77.1)	64 (77.1)	
*Site*			*< 0.001*
Pelvis	36 (43.4)	37 (44.6)	
Upper calyx	5 (6.0)	14 (16.9)	
Middle calyx	20 (24.1)	11 (13.3)	
Complex	3 (3.6)	2 (2.4)	
Multiple stones, n (%)	3 (3.6)	2 (2.4)	> 0.999
Radiopaque stones, n (%)	72 (86.7)	71 (58.5)	0.822
Stone radio-density (HU), median (IQR)	1200 (950–1300)	785 (650–1200)	0.001
Stone radio-density, n (%)			0.008
≤ 1000 HU	32 (38.6)	49 (59.0)	
> 1000 HU	51 (61.4)	34 (41.0)	
Present, n (%)	25 (30.1)	15 (18.1)	0.070

RIRS was performed under general anesthesia in 38.6% and spinal anesthesia in 61.4% of cases. Renal access failure occurred in 15 cases (18.1%), requiring pre-stenting and delayed intervention. Seven procedures (8.4%) were aborted due to prolonged operative time. Among the 83 patients, 16 underwent staged procedures: 11 required two sessions, and 5 required three sessions. SWL was performed under analgesia or sedation in all cases, with no premature procedural terminations. A total of 26.5% of patients required two SWL sessions, and 59.0% required three. Operative time was significantly longer in the RIRS group (*p* = 0.009), while fluoroscopy time was significantly lower (*p* < 0.001). Procedural details are shown in Table 2.

Initial SFR was significantly higher in the RIRS group compared to SWL (72.3% vs. 56.6%; *p* = 0.035). After completion of staged procedures, the cumulative SFR for RIRS increased to 81.9% (*p* < 0.001). Stratified analysis by anomaly type showed a significantly higher SFR for RIRS in patients with simple malrotation (*p* = 0.004); no significant difference was noted in other anomaly subgroups. However, subgroup analysis was limited by small sample sizes.

In the RIRS group, a statistically significant but clinically insignificant reduction in haemoglobin was observed (mean change − 0.5 g/dL, SE 0.02; *p* < 0.001), with no patients requiring transfusion. The median hospital stay for RIRS was 2 days (IQR 1.0); all SWL procedures were performed on an outpatient basis. Unscheduled rehospitalization occurred in 6.0% of RIRS patients (all for pyelonephritis) and 7.2% of SWL patients (for renal pain, hematuria, or pyelonephritis; *p* > 0.999).

**Table 2:** Perioperative data and outcome

Variables	RIRS group (*n* = 83)	SWL group (*n* = 83)	Diff (95% CI)	*p*-value
Operative time, min.,				< 0.001
Mean (SD)	71.8 (13.6)	38.9 (7.8)	−32.9 (29.5 – −36.3)	
Median (IQR)	71.0 (62.5–84.0)	39.0 (33.3–44.0)		
Fluoroscopy time, sec., median (IQR)				< 0.001
Mean (SD)	52.1 (59.1)	108.0 (42.6)	−55.9 (−71.7 – −40.1)	
Median (IQR)	26.0 (23.3–63.5)	96.0 (90.0–102.0)		
Retreatment, n (%) 2 sessions 3 sessions	16 (19.3) 11 5	71 (85.5) 22 49	66.3 (−77.6 – −54.9)	< 0.001
SFR, n (%)	68 (81.9)	47 (56.6)	25.3 (11.8–38.8)	< 0.001
*According to renal anomalies*				
Malrotated	39/46 (84.8)	28/46 (60.9)		0.010
Horseshoe	10/13 (76.9)	7/13 (53.8)		0.216
Ectopic	14/19 (73.7)	9/19 (47.4)		0.097
Duplicated	5/5 (100)	3/5 (60.0)		0.444
Unscheduled rehospitalization, n (%)	5 (7.5)	6 (8.4)	1.2 (−8.8–6.4)	0.755
Complications, n (%) *MCC grade (3*,* 4)*	20 (24.1) 3 (3.6)	17 (20.5) 2 (2.4)	3.6 (−9.0–16.3)	0.576 > 0.999
Direct medical cost (EGP)				< 0.001
Mean (SD)	101,812 (46,617)	49,642 (30,223)	52,170 (40,114–64,226)	
Median (IQR)	77,762 (66,767–107770)	42,756 (22,756–79,771)		

Overall complication rates were comparable between groups (RIRS 24.1% vs. SWL 20.5%; *p* = 0.576). In the RIRS group, the most frequent complications were postoperative fever (19.3%) and lower urinary tract symptoms (LUTS) (15.7%). In the SWL group, renal pain and transient haematuria were the most common (each 9.6%). Perioperative complications stratified by modified Clavien–Dindo classification are detailed in Table 3.

**Table 3:** Intra- and post-operative complications

Complications	MMC grade	Number	%
*RIRS group*			
Intraoperative		3	3.6
Ureteral injury Subcapsular hematoma	3b 3b	2 1	2.4 1.2
Postoperative		20	24.1
Post-operative fever	1	16	19.3
Clinically significant LUTS	1	13	15.7
Pyelonephritis	2	5	6.0
Transient hematuria	1	5	6.0
*SWL group*			
Intraoperative		0	0
Postoperative		17	20.5
Hematuria	1	8	9.6
Clinically significant LUTS	1	7	8.4
Pyelonephritis	2	1	1.2
Steinstrasse	3b	2	2.4
Renal pain	1	8	9.6

*LUTS*, Lower urinary tract symptoms; *MMC*, Modified Clavien classification; *UTI*, Urinary tract infection

## Discussion

Treatment of renal stones in patients with congenital renal anomalies poses a significant challenge in urology. Abnormal renal anatomy complicates conventional treatment approaches, such as open surgery or PCNL, often leading to increased morbidity [5, 14, 15]. As a result, selecting the most appropriate treatment modality is crucial for achieving optimal outcomes.

In this study, we compared the safety and efficacy of two minimally invasive techniques, RIRS and SWL, for treating renal stones in patients with congenital renal anomalies. We used matched cases to minimize potential biases and ensure a more reliable comparison.

Our findings revealed that RIRS achieved a significantly higher SFR than SWL (81.9% vs. 56.6%, *p* < 0.001). While both procedures had relatively high complication rates (24.1% for RIRS vs. 20.5% for SWL), the difference was insignificant, and most complications were minor and self-limiting. Interestingly, RIRS required significantly less fluoroscopy time but was associated with a longer hospital stay. These results align with prior studies, reinforcing RIRS as a more effective treatment option in patients with complex renal anatomy.

One of the most significant findings was the superior single-session SFR achieved with RIRS. This concurs with earlier reports. Gokce et al. [10] reported SFRs of 73.9% for RIRS and 47.7% for SWL in anomalous kidneys. Similarly, Kartal et al. [16] noted a single-session SFR of 71.4% for RIRS, increasing to 85.7% after staged interventions. Ugurlu et al. [17] also documented high overall SFRs of 88% for RIRS, including staged cases.

The higher SFR observed with RIRS may be attributed to its direct visual access to the collecting system and the ability to treat multiple stones in a single session. Moreover, the use of a flexible ureteroscope allows for navigation through anomalous renal configurations, which is particularly advantageous in cases of malrotation, ectopia, or fusion anomalies. In our series, patients with simple malrotation had significantly better outcomes with RIRS than with SWL, while the differences were less pronounced in patients with other renal anomalies. However, the subgroup analysis was limited by the small sample sizes within each anomaly category.

By contrast, the effectiveness of SWL in patients with congenital renal anomalies remains inconsistent. In our study, SWL achieved a stone-free rate of only 56.6%, even after up to three treatment sessions. This is substantially lower than the rates typically reported in patients with normal renal anatomy [18], likely due to anatomical factors that hinder accurate stone localization and fragment clearance. Reported SFRs for SWL in anomalous kidneys vary widely across studies [19–22], which may be attributed to differences in the types of anomalies included, as well as variations in patient and stone characteristics. The relatively lower SFR observed in our cohort may also be explained by the high prevalence of radiodense stones, which are known to be less responsive to SWL. Interestingly, patients treated with RIRS had significantly higher stone radiodensity (*p* = 0.001), yet achieved better outcomes, supporting the efficacy of RIRS in managing stones in the setting of both anatomical abnormalities and unfavorable stone characteristics.

A notable drawback of SWL is the frequent need for repeated sessions. Our patients underwent an average of 2.5 SWL sessions, consistent with prior findings suggesting SWL may be inadequate in complex anatomies [8, 9]. In contrast, although 19.3% of RIRS patients required re-intervention, this was significantly lower than the 85.5% retreatment rate observed in the SWL group.

Despite the longer operative time for RIRS (median: 71.0 min vs. 39.0 min for SWL, *p* < 0.001), it required significantly less fluoroscopy (median: 26.0 vs. 96.0 s, *p* < 0.001). This reduction in radiation exposure is particularly advantageous in patients needing repeated imaging or procedures. Gokce et al. [10] similarly reported reduced fluoroscopy use with RIRS, reinforcing its safety benefits.

Length of hospitalization also differed. RIRS patients had a median hospital stay of 2 days, while SWL was performed entirely on an outpatient basis. This increased hospitalization is attributable to RIRS being more invasive. However, the trade-off may be justified given RIRS’s superior SFR and reduced need for further interventions, potentially translating to fewer future hospital visits and procedures.

Complication rates were similar between the two groups, with most being mild and transient. In the RIRS group, common complications included transient fever (19.3%) and LUTS (15.7%). In the SWL group, the most frequently reported complications were transient hematuria and renal colic (both at 9.6%). The type and frequency of complications observed in our study are consistent with those reported in previous studies involving both anomalous and anatomically normal kidneys [5, 10, 23].

Cost considerations play a crucial role, particularly in settings with limited resources. In our analysis, RIRS was associated with a higher initial direct cost (EGP 77,762 vs. EGP 42,756 for SWL), primarily due to the requirement for anesthesia, costly disposable equipment, and inpatient hospitalization. However, the superior efficacy of RIRS and its significantly lower retreatment rates may translate into greater cost-effectiveness in the long term. Conversely, although SWL offers a lower upfront cost, its reduced efficacy in patients with complex renal anatomy often necessitates multiple sessions and additional interventions, potentially increasing the overall treatment cost.

This study has several limitations. The heterogeneity of congenital anomalies in our sample may limit generalizability. Additionally, RIRS procedures were conducted by highly experienced urologists at a tertiary center, which may not reflect outcomes in less specialized settings. The retrospective design of the SWL group introduces potential selection bias, and the relatively short follow-up may not capture late complications or stone recurrence. Future prospective, randomized studies with longer follow-up and more homogeneous populations are warranted to validate and expand on our findings.

## Conclusion

RIRS demonstrates superior efficacy with acceptable complication rates compared to SWL in treating renal stones in patients with congenital renal anomalies. Its higher SFR, fewer retreatments, and reduced radiation exposure support its use as a preferred modality in this complex patient group. Nevertheless, treatment should be tailored to individual patient characteristics, anatomy, and institutional resources to optimize outcomes.

## Data Availability

The datasets used and/or analyzed during the current study are available from the corresponding author upon reasonable request.
